# Supplementary material for: Study on the Interactions of Cyclins with CDKs Involved in Auxin Signal during Leaf Development by WGCNA in Populus alba
Source: Int J Mol Sci. 2023 Aug 30;24(17):13445. doi: 10.3390/ijms241713445 (PMC10487486; doi:10.3390/ijms241713445)
Supplement: Supplementary file 1 [file ijms-24-13445-s001.zip › Supplementary Files/Figure S3. Cis-acting elements of cyclin and CDK genes in promoters.pdf]

| | Auxin | | | MeJA | | GA | | | SA | ABA | Light | | | | | | | | | | Division | | Zein | Flavonoid | | Cir | Abiotic and biotic stresses | | | | | |
|---|---|---|---|---|---|---|---|---|---|---|---|---|---|---|---|---|---|---|---|---|---|---|---|---|---|---|---|---|---|---|---|---|
| | AUXREs | TGA-element | AuxRR-core | CGTCA-motif | TGACG-motif | GARE-motif | P-box | TATC-box | TCA-element | ABRE | Box 4 | GATA-motif | TCT-motif | GT1-motif | ATCT-motif | TCCC-motif | I-box | AE-box | AT1-motif | MRE | MSA-like | CAT-box | O2-site | CCAAT-box | MBSI | circadian | ARE | W box | TC-rich repeats | G-box | MBS | LTR |
| PoalbCYCA1;1 | 0 | 0 | 0 | 1 | 1 | 0 | 1 | 0 | 2 | 1 | 1 | 0 | 1 | 1 | 0 | 0 | 1 | 2 | 0 | 1 | 2 | 0 | 1 | 1 | 0 | 0 | 3 | 0 | 1 | 2 | 1 | 0 |
| PoalbCYCA1;2 | 2 | 0 | 0 | 2 | 2 | 0 | 1 | 0 | 1 | 2 | 0 | 0 | 0 | 2 | 0 | 0 | 0 | 2 | 0 | 0 | 3 | 0 | 1 | 1 | 0 | 0 | 4 | 0 | 0 | 3 | 0 | 0 |
| PoalbCYCA2;1 | 2 | 1 | 0 | 0 | 0 | 0 | 0 | 0 | 0 | 4 | 1 | 0 | 0 | 2 | 1 | 1 | 0 | 1 | 0 | 0 | 0 | 0 | 0 | 0 | 0 | 1 | 0 | 0 | 0 | 3 | 1 | 3 |
| PoalbCYCA2;2 | 1 | 3 | 0 | 0 | 0 | 0 | 1 | 0 | 0 | 3 | 3 | 0 | 1 | 2 | 0 | 1 | 0 | 1 | 0 | 0 | 0 | 0 | 2 | 0 | 0 | 0 | 2 | 0 | 1 | 1 | 1 | 1 |
| PoalbCYCA2;3 | 0 | 1 | 0 | 2 | 2 | 0 | 0 | 0 | 4 | 0 | 0 | 0 | 1 | 0 | 0 | 0 | 0 | 3 | 0 | 0 | 0 | 0 | 1 | 0 | 0 | 0 | 7 | 0 | 0 | 1 | 0 | 1 |
| PoalbCYCA2;4 | 0 | 1 | 0 | 1 | 1 | 0 | 1 | 0 | 2 | 1 | 0 | 2 | 1 | 0 | 0 | 0 | 1 | 2 | 0 | 0 | 0 | 2 | 0 | 0 | 0 | 1 | 1 | 2 | 0 | 1 | 1 | 1 |
| PoalbCYCA3;1 | 0 | 0 | 0 | 0 | 0 | 1 | 0 | 0 | 1 | 2 | 0 | 1 | 3 | 2 | 0 | 1 | 2 | 0 | 1 | 1 | 0 | 0 | 0 | 0 | 0 | 0 | 1 | 2 | 0 | 2 | 0 | 0 |
| PoalbCYCA3;2 | 0 | 0 | 0 | 0 | 0 | 0 | 0 | 0 | 1 | 2 | 8 | 0 | 0 | 1 | 0 | 0 | 1 | 0 | 0 | 0 | 0 | 1 | 1 | 0 | 0 | 0 | 1 | 1 | 3 | 1 | 2 | 0 |
| PoalbCYCA3;3 | 0 | 0 | 0 | 1 | 1 | 0 | 0 | 0 | 1 | 2 | 7 | 0 | 0 | 3 | 0 | 1 | 1 | 0 | 1 | 0 | 0 | 1 | 1 | 0 | 0 | 0 | 1 | 2 | 1 | 1 | 2 | 0 |
| PoalbCYCB1;1 | 1 | 0 | 0 | 0 | 0 | 0 | 1 | 1 | 0 | 2 | 4 | 1 | 2 | 0 | 0 | 1 | 1 | 0 | 0 | 0 | 2 | 0 | 0 | 2 | 0 | 0 | 1 | 1 | 0 | 1 | 0 | 0 |
| PoalbCYCB1;2 | 0 | 0 | 0 | 0 | 0 | 0 | 0 | 0 | 0 | 1 | 6 | 0 | 1 | 0 | 1 | 1 | 1 | 1 | 1 | 1 | 2 | 1 | 0 | 2 | 0 | 0 | 2 | 0 | 1 | 2 | 0 | 0 |
| PoalbCYCB1;3 | 1 | 0 | 0 | 3 | 3 | 0 | 1 | 0 | 0 | 0 | 6 | 1 | 0 | 3 | 0 | 1 | 0 | 0 | 2 | 1 | 2 | 1 | 0 | 2 | 0 | 0 | 5 | 2 | 0 | 0 | 0 | 0 |
| PoalbCYCB1;4 | 0 | 0 | 0 | 1 | 1 | 0 | 0 | 0 | 2 | 3 | 4 | 1 | 1 | 2 | 0 | 0 | 0 | 1 | 0 | 2 | 2 | 0 | 2 | 1 | 0 | 0 | 4 | 1 | 3 | 4 | 0 | 1 |
| PoalbCYCB2;1 | 2 | 1 | 0 | 1 | 1 | 0 | 1 | 0 | 1 | 1 | 2 | 0 | 1 | 1 | 1 | 1 | 0 | 0 | 0 | 0 | 2 | 0 | 0 | 3 | 0 | 0 | 1 | 0 | 0 | 1 | 0 | 0 |
| PoalbCYCB2;2 | 1 | 0 | 0 | 2 | 2 | 0 | 1 | 0 | 0 | 1 | 3 | 1 | 0 | 2 | 0 | 2 | 2 | 1 | 0 | 1 | 1 | 0 | 0 | 2 | 0 | 0 | 4 | 0 | 0 | 1 | 1 | 0 |
| PoalbCYCB2;3 | 1 | 0 | 0 | 1 | 1 | 0 | 0 | 0 | 3 | 1 | 2 | 1 | 0 | 2 | 1 | 0 | 0 | 2 | 0 | 0 | 1 | 1 | 0 | 2 | 0 | 0 | 2 | 1 | 1 | 0 | 0 | 0 |
| PoalbCYCB3;1 | 1 | 0 | 0 | 2 | 2 | 0 | 0 | 0 | 1 | 2 | 5 | 4 | 1 | 2 | 0 | 0 | 0 | 1 | 0 | 1 | 0 | 0 | 0 | 1 | 0 | 0 | 2 | 0 | 1 | 2 | 0 | 0 |
| PoalbCYCC1;1 | 0 | 0 | 0 | 0 | 0 | 1 | 2 | 1 | 0 | 0 | 3 | 1 | 1 | 1 | 1 | 0 | 2 | 0 | 0 | 0 | 0 | 0 | 0 | 1 | 0 | 0 | 2 | 1 | 1 | 0 | 1 | 0 |
| PoalbCYCC1;2 | 0 | 1 | 0 | 1 | 1 | 0 | 0 | 0 | 0 | 1 | 4 | 0 | 0 | 5 | 0 | 0 | 0 | 0 | 0 | 2 | 0 | 0 | 0 | 0 | 0 | 0 | 5 | 2 | 0 | 0 | 2 | 3 |
| PoalbCYCD1;1 | 2 | 0 | 0 | 0 | 0 | 0 | 0 | 0 | 1 | 2 | 3 | 0 | 3 | 2 | 0 | 0 | 0 | 1 | 0 | 0 | 0 | 1 | 1 | 1 | 0 | 0 | 0 | 0 | 0 | 4 | 1 | 0 |
| PoalbCYCD1;2 | 1 | 0 | 0 | 0 | 0 | 0 | 0 | 0 | 0 | 0 | 2 | 0 | 0 | 3 | 0 | 0 | 0 | 2 | 1 | 1 | 0 | 0 | 0 | 0 | 0 | 0 | 3 | 2 | 0 | 0 | 1 | 1 |
| PoalbCYCD1;3 | 2 | 0 | 0 | 0 | 0 | 0 | 0 | 0 | 0 | 5 | 1 | 0 | 0 | 3 | 0 | 1 | 0 | 1 | 0 | 0 | 0 | 0 | 0 | 1 | 0 | 0 | 2 | 2 | 0 | 5 | 1 | 1 |
| PoalbCYCD1;4 | 2 | 0 | 0 | 1 | 1 | 0 | 0 | 0 | 0 | 2 | 3 | 2 | 2 | 2 | 0 | 1 | 1 | 4 | 2 | 0 | 0 | 2 | 0 | 1 | 0 | 0 | 2 | 2 | 0 | 2 | 0 | 0 |
| PoalbCYCD1;5 | 1 | 1 | 0 | 2 | 2 | 0 | 0 | 0 | 0 | 2 | 3 | 0 | 0 | 5 | 0 | 1 | 0 | 1 | 0 | 2 | 0 | 1 | 0 | 0 | 0 | 0 | 4 | 2 | 0 | 3 | 0 | 2 |
| PoalbCYCD1;6 | 0 | 0 | 0 | 2 | 2 | 2 | 0 | 1 | 0 | 1 | 2 | 0 | 3 | 1 | 1 | 0 | 0 | 0 | 0 | 0 | 0 | 0 | 1 | 0 | 0 | 0 | 0 | 0 | 0 | 1 | 1 | 0 |
| PoalbCYCD2;1 | 0 | 0 | 0 | 1 | 1 | 0 | 1 | 0 | 0 | 0 | 3 | 1 | 0 | 0 | 0 | 1 | 0 | 0 | 1 | 0 | 0 | 0 | 0 | 0 | 0 | 0 | 1 | 0 | 0 | 0 | 0 | 2 |
| PoalbCYCD2;2 | 0 | 0 | 0 | 1 | 1 | 0 | 0 | 1 | 0 | 1 | 4 | 1 | 0 | 6 | 0 | 0 | 0 | 0 | 2 | 0 | 0 | 0 | 1 | 0 | 0 | 0 | 3 | 0 | 0 | 1 | 1 | 1 |
| PoalbCYCD3;1 | 2 | 1 | 0 | 2 | 2 | 0 | 0 | 0 | 3 | 0 | 1 | 0 | 1 | 1 | 0 | 0 | 0 | 0 | 0 | 1 | 0 | 0 | 2 | 0 | 0 | 0 | 3 | 1 | 1 | 1 | 1 | 2 |
| PoalbCYCD3;2 | 2 | 0 | 0 | 0 | 0 | 0 | 0 | 0 | 0 | 2 | 1 | 1 | 1 | 1 | 0 | 0 | 1 | 0 | 1 | 1 | 1 | 2 | 0 | 0 | 0 | 0 | 3 | 0 | 0 | 2 | 1 | 1 |
| PoalbCYCD3;3 | 1 | 0 | 0 | 3 | 3 | 0 | 0 | 0 | 1 | 1 | 7 | 1 | 0 | 0 | 1 | 1 | 0 | 0 | 1 | 0 | 0 | 1 | 0 | 0 | 0 | 0 | 1 | 1 | 0 | 0 | 0 | 0 |
| PoalbCYCD3;4 | 0 | 0 | 0 | 0 | 0 | 0 | 0 | 0 | 0 | 3 | 6 | 1 | 0 | 3 | 0 | 1 | 0 | 0 | 0 | 1 | 0 | 0 | 0 | 0 | 0 | 0 | 1 | 0 | 0 | 4 | 0 | 0 |
| PoalbCYCD3;5 | 2 | 1 | 0 | 2 | 2 | 0 | 0 | 0 | 0 | 1 | 3 | 1 | 2 | 2 | 0 | 0 | 1 | 0 | 1 | 1 | 0 | 0 | 0 | 1 | 0 | 0 | 1 | 0 | 1 | 1 | 0 | 0 |
| PoalbCYCD5;1 | 1 | 0 | 0 | 0 | 0 | 0 | 0 | 0 | 0 | 3 | 4 | 2 | 1 | 5 | 0 | 0 | 1 | 2 | 0 | 1 | 0 | 1 | 0 | 0 | 1 | 0 | 2 | 0 | 1 | 3 | 0 | 0 |
| PoalbCYCD5;2 | 3 | 1 | 0 | 3 | 3 | 1 | 0 | 1 | 0 | 2 | 4 | 0 | 0 | 2 | 0 | 0 | 0 | 1 | 0 | 0 | 0 | 0 | 0 | 0 | 0 | 0 | 1 | 2 | 0 | 4 | 0 | 0 |
| PoalbCYCD5;3 | 0 | 0 | 0 | 2 | 2 | 0 | 0 | 0 | 0 | 1 | 2 | 2 | 0 | 0 | 0 | 1 | 0 | 0 | 0 | 1 | 0 | 1 | 3 | 0 | 0 | 0 | 1 | 0 | 0 | 2 | 1 | 0 |
| PoalbCYCD6;1 | 1 | 0 | 0 | 0 | 0 | 1 | 2 | 0 | 0 | 0 | 6 | 0 | 0 | 5 | 0 | 0 | 1 | 2 | 1 | 0 | 0 | 0 | 0 | 0 | 0 | 1 | 2 | 0 | 1 | 0 | 1 | 0 |
| PoalbCYCD6;2 | 0 | 0 | 0 | 1 | 1 | 0 | 1 | 0 | 1 | 0 | 4 | 1 | 0 | 3 | 0 | 1 | 0 | 0 | 0 | 0 | 0 | 0 | 0 | 0 | 0 | 0 | 3 | 1 | 0 | 0 | 1 | 0 |
| PoalbCYCD6;3 | 0 | 0 | 0 | 0 | 0 | 0 | 2 | 0 | 1 | 0 | 5 | 1 | 1 | 3 | 0 | 0 | 0 | 0 | 1 | 0 | 0 | 0 | 0 | 0 | 2 | 0 | 3 | 4 | 0 | 0 | 0 | 0 |
| PoalbCYCD6;4 | 1 | 0 | 0 | 0 | 0 | 0 | 2 | 1 | 0 | 3 | 6 | 0 | 2 | 0 | 0 | 0 | 0 | 0 | 1 | 0 | 0 | 0 | 0 | 0 | 0 | 0 | 1 | 2 | 3 | 2 | 0 | 0 |
| PoalbCYCD6;5 | 0 | 0 | 0 | 0 | 0 | 0 | 0 | 0 | 0 | 6 | 3 | 0 | 0 | 2 | 0 | 0 | 0 | 2 | 0 | 2 | 0 | 2 | 1 | 1 | 0 | 0 | 3 | 1 | 0 | 4 | 0 | 0 |
| PoalbCYCD7;1 | 0 | 0 | 0 | 0 | 0 | 0 | 0 | 0 | 0 | 8 | 6 | 1 | 1 | 2 | 0 | 1 | 0 | 0 | 0 | 1 | 0 | 0 | 0 | 0 | 0 | 0 | 0 | 1 | 0 | 9 | 0 | 0 |
| PoalbCYCH1;1 | 0 | 0 | 0 | 0 | 0 | 0 | 1 | 0 | 0 | 3 | 5 | 1 | 2 | 4 | 1 | 0 | 0 | 0 | 1 | 0 | 0 | 0 | 1 | 0 | 0 | 0 | 1 | 1 | 0 | 2 | 0 | 0 |
| PoalbCYCL1;1 | 0 | 0 | 0 | 1 | 1 | 0 | 0 | 1 | 1 | 4 | 4 | 2 | 0 | 4 | 3 | 0 | 0 | 0 | 1 | 0 | 0 | 0 | 0 | 0 | 0 | 0 | 3 | 0 | 0 | 7 | 0 | 2 |
| PoalbCYCT1;1 | 1 | 0 | 0 | 1 | 1 | 0 | 1 | 0 | 0 | 1 | 4 | 2 | 0 | 1 | 1 | 0 | 0 | 1 | 0 | 0 | 0 | 1 | 0 | 0 | 0 | 0 | 5 | 0 | 2 | 1 | 0 | 0 |
| PoalbCYCT1;2 | 0 | 0 | 0 | 2 | 2 | 0 | 0 | 0 | 1 | 2 | 2 | 1 | 0 | 3 | 1 | 0 | 2 | 1 | 0 | 0 | 0 | 0 | 0 | 0 | 1 | 0 | 3 | 1 | 0 | 2 | 0 | 1 |
| PoalbCYCT1;3 | 1 | 0 | 0 | 2 | 2 | 0 | 1 | 0 | 0 | 6 | 6 | 2 | 1 | 1 | 0 | 0 | 0 | 2 | 0 | 1 | 1 | 0 | 0 | 1 | 0 | 0 | 0 | 0 | 0 | 7 | 0 | 2 |
| PoalbCYCT1;4 | 0 | 1 | 0 | 2 | 2 | 1 | 0 | 0 | 1 | 2 | 4 | 0 | 2 | 1 | 0 | 0 | 1 | 0 | 0 | 0 | 0 | 0 | 1 | 0 | 0 | 0 | 5 | 0 | 0 | 2 | 0 | 1 |
| PoalbCYCT1;5 | 3 | 1 | 0 | 0 | 0 | 2 | 0 | 1 | 0 | 1 | 1 | 0 | 2 | 4 | 0 | 1 | 0 | 0 | 1 | 2 | 0 | 1 | 1 | 0 | 0 | 0 | 3 | 1 | 2 | 1 | 0 | 0 |
| PoalbCYCT1;6 | 2 | 2 | 0 | 0 | 0 | 0 | 0 | 0 | 0 | 0 | 2 | 0 | 1 | 3 | 1 | 0 | 1 | 1 | 0 | 2 | 0 | 0 | 1 | 0 | 0 | 0 | 0 | 2 | 1 | 0 | 2 | 0 |
| PoalbCYCU1;1 | 0 | 0 | 1 | 1 | 1 | 0 | 0 | 0 | 0 | 4 | 1 | 0 | 0 | 3 | 0 | 0 | 0 | 0 | 0 | 1 | 0 | 1 | 2 | 0 | 0 | 0 | 0 | 1 | 0 | 5 | 0 | 1 |
| PoalbCYCU2;1 | 1 | 1 | 1 | 3 | 3 | 0 | 0 | 0 | 0 | 5 | 5 | 0 | 1 | 2 | 0 | 2 | 0 | 0 | 1 | 0 | 0 | 0 | 0 | 0 | 0 | 0 | 1 | 0 | 0 | 6 | 1 | 1 |
| PoalbCYCU2;2 | 3 | 1 | 0 | 1 | 1 | 1 | 0 | 0 | 3 | 0 | 11 | 0 | 0 | 0 | 0 | 1 | 0 | 0 | 0 | 0 | 0 | 0 | 1 | 0 | 0 | 0 | 4 | 0 | 0 | 0 | 0 | 0 |
| PoalbCYCU3;1 | 1 | 0 | 0 | 0 | 0 | 1 | 0 | 0 | 1 | 2 | 2 | 1 | 1 | 2 | 0 | 2 | 1 | 0 | 0 | 0 | 0 | 0 | 0 | 0 | 1 | 1 | 2 | 0 | 2 | 2 | 0 | 0 |
| PoalbCYCU3;2 | 1 | 0 | 0 | 2 | 2 | 0 | 0 | 0 | 0 | 0 | 3 | 1 | 0 | 2 | 0 | 0 | 0 | 1 | 0 | 3 | 0 | 0 | 0 | 0 | 0 | 0 | 2 | 2 | 0 | 0 | 2 | 0 |
| PoalbCYCU3;3 | 0 | 1 | 0 | 0 | 0 | 1 | 1 | 1 | 0 | 1 | 4 | 0 | 3 | 3 | 0 | 0 | 0 | 0 | 0 | 0 | 0 | 0 | 1 | 0 | 0 | 0 | 0 | 0 | 0 | 1 | 2 | 1 |
| PoalbCYCU3;4 | 0 | 1 | 0 | 0 | 0 | 0 | 0 | 0 | 1 | 4 | 5 | 1 | 1 | 3 | 0 | 0 | 0 | 1 | 0 | 1 | 0 | 0 | 0 | 0 | 0 | 0 | 3 | 0 | 1 | 5 | 1 | 0 |
| PoalbCYCU4;1 | 2 | 0 | 0 | 0 | 0 | 1 | 1 | 0 | 0 | 2 | 5 | 0 | 0 | 6 | 0 | 0 | 0 | 0 | 0 | 0 | 0 | 0 | 1 | 0 | 0 | 1 | 2 | 1 | 1 | 3 | 0 | 2 |
| PoalbCYCU4;2 | 2 | 1 | 0 | 0 | 0 | 1 | 1 | 0 | 0 | 3 | 5 | 0 | 0 | 8 | 0 | 0 | 0 | 0 | 0 | 0 | 0 | 0 | 1 | 0 | 0 | 1 | 2 | 1 | 1 | 4 | 0 | 2 |
| PoalbCYCU4;3 | 0 | 1 | 0 | 0 | 0 | 0 | 1 | 0 | 0 | 1 | 5 | 0 | 0 | 3 | 0 | 0 | 0 | 2 | 0 | 0 | 0 | 0 | 0 | 0 | 0 | 0 | 2 | 0 | 1 | 2 | 0 | 1 |
| PoalbCYCU4;4 | 0 | 0 | 0 | 1 | 1 | 2 | 0 | 0 | 0 | 3 | 2 | 2 | 2 | 0 | 0 | 1 | 0 | 1 | 0 | 0 | 0 | 1 | 1 | 0 | 0 | 2 | 5 | 0 | 0 | 3 | 1 | 0 |
| PoalbCYCU4;5 | 0 | 0 | 0 | 1 | 1 | 2 | 0 | 0 | 0 | 2 | 1 | 1 | 2 | 0 | 0 | 1 | 1 | 1 | 0 | 0 | 0 | 1 | 1 | 0 | 0 | 1 | 4 | 0 | 0 | 2 | 1 | 0 |
| PoalbCYCU4;6 | 0 | 0 | 0 | 1 | 1 | 2 | 0 | 0 | 0 | 4 | 1 | 2 | 1 | 2 | 0 | 1 | 1 | 1 | 0 | 0 | 0 | 1 | 1 | 0 | 0 | 1 | 7 | 0 | 0 | 4 | 0 | 0 |
| PoalbSDS | 1 | 1 | 2 | 0 | 0 | 1 | 0 | 0 | 0 | 5 | 8 | 0 | 1 | 0 | 0 | 2 | 0 | 0 | 0 | 1 | 0 | 0 | 0 | 0 | 0 | 0 | 2 | 2 | 0 | 6 | 0 | 1 |
| PoalbCDKA;1 | 0 | 0 | 0 | 1 | 1 | 0 | 0 | 0 | 1 | 0 | 4 | 1 | 2 | 1 | 1 | 1 | 0 | 0 | 0 | 0 | 0 | 0 | 0 | 0 | 1 | 0 | 0 | 3 | 0 | 0 | 1 | 0 |
| PoalbCDKB1;1 | 0 | 0 | 0 | 0 | 0 | 0 | 0 | 0 | 1 | 0 | 2 | 1 | 0 | 1 | 1 | 1 | 0 | 0 | 0 | 0 | 1 | 0 | 1 | 1 | 0 | 0 | 2 | 3 | 0 | 0 | 2 | 0 |
| PoalbCDKB1;2 | 0 | 0 | 0 | 0 | 0 | 1 | 0 | 0 | 0 | 1 | 4 | 2 | 0 | 2 | 0 | 3 | 0 | 3 | 1 | 1 | 0 | 1 | 0 | 2 | 1 | 1 | 3 | 0 | 1 | 2 | 0 | 1 |
| PoalbCDKB2;1 | 2 | 0 | 0 | 0 | 0 | 1 | 1 | 0 | 1 | 0 | 1 | 3 | 2 | 2 | 1 | 0 | 0 | 0 | 0 | 2 | 0 | 1 | 0 | 1 | 0 | 0 | 6 | 1 | 0 | 0 | 3 | 0 |
| PoalbCDKB2;2 | 0 | 0 | 2 | 1 | 1 | 0 | 0 | 0 | 0 | 2 | 1 | 2 | 1 | 1 | 2 | 1 | 0 | 0 | 0 | 0 | 0 | 0 | 1 | 0 | 0 | 0 | 1 | 0 | 1 | 3 | 1 | 0 |
| PoalbCDKC;1 | 4 | 0 | 0 | 1 | 1 | 0 | 1 | 0 | 0 | 3 | 2 | 1 | 0 | 2 | 0 | 0 | 0 | 2 | 0 | 0 | 0 | 0 | 0 | 0 | 0 | 0 | 4 | 1 | 0 | 4 | 0 | 1 |
| PoalbCDKC;2 | 3 | 0 | 0 | 2 | 2 | 0 | 0 | 0 | 0 | 2 | 0 | 0 | 0 | 0 | 0 | 0 | 0 | 0 | 0 | 1 | 0 | 2 | 2 | 0 | 0 | 1 | 1 | 2 | 0 | 2 | 2 | 0 |
| PoalbCDKD;1 | 0 | 0 | 2 | 0 | 0 | 0 | 0 | 0 | 1 | 0 | 5 | 1 | 0 | 3 | 1 | 0 | 0 | 0 | 0 | 1 | 0 | 0 | 0 | 0 | 0 | 0 | 3 | 2 | 0 | 0 | 0 | 1 |
| PoalbCDKD;3 | 0 | 0 | 0 | 0 | 0 | 1 | 0 | 0 | 1 | 6 | 3 | 5 | 0 | 0 | 0 | 0 | 6 | 0 | 0 | 0 | 0 | 0 | 0 | 0 | 0 | 0 | 1 | 0 | 1 | 6 | 0 | 0 |
| PoalbCDKE;1 | 1 | 0 | 1 | 0 | 0 | 0 | 1 | 0 | 0 | 0 | 1 | 1 | 1 | 1 | 0 | 0 | 0 | 0 | 0 | 0 | 0 | 0 | 1 | 1 | 0 | 1 | 1 | 1 | 0 | 0 | 2 | 0 |
| PoalbCDKE;2 | 0 | 0 | 0 | 2 | 2 | 1 | 0 | 0 | 0 | 1 | 1 | 2 | 3 | 1 | 1 | 1 | 0 | 0 | 0 | 1 | 0 | 0 | 0 | 0 | 0 | 1 | 2 | 1 | 1 | 1 | 0 | 0 |
| PoalbCDKF;1 | 2 | 0 | 0 | 3 | 3 | 2 | 1 | 0 | 0 | 2 | 0 | 0 | 3 | 1 | 0 | 1 | 0 | 0 | 0 | 0 | 0 | 0 | 3 | 0 | 0 | 0 | 3 | 2 | 1 | 2 | 2 | 0 |
| PoalbCDKG;1 | 1 | 0 | 0 | 0 | 0 | 1 | 0 | 0 | 3 | 3 | 0 | 0 | 1 | 0 | 0 | 1 | 0 | 0 | 0 | 2 | 0 | 0 | 1 | 1 | 0 | 0 | 4 | 2 | 0 | 1 | 0 | 0 |
| PoalbCDKG;2 | 0 | 0 | 1 | 0 | 0 | 0 | 2 | 1 | 0 | 4 | 2 | 1 | 1 | 3 | 1 | 0 | 0 | 0 | 0 | 0 | 0 | 0 | 1 | 0 | 0 | 1 | 1 | 1 | 1 | 2 | 1 | 2 |
| PoalbCDKG;3 | 1 | 0 | 0 | 0 | 0 | 0 | 0 | 1 | 0 | 2 | 2 | 0 | 1 | 2 | 0 | 0 | 0 | 1 | 1 | 1 | 0 | 1 | 0 | 0 | 1 | 0 | 1 | 0 | 0 | 2 | 2 | 0 |
| PoalbCDKG;4 | 2 | 0 | 0 | 2 | 2 | 0 | 3 | 0 | 0 | 0 | 8 | 0 | 1 | 0 | 0 | 0 | 0 | 0 | 0 | 0 | 0 | 1 | 0 | 1 | 0 | 0 | 2 | 1 | 0 | 1 | 2 | 0 |
| PoalbCDKG;5 | 1 | 0 | 0 | 3 | 3 | 0 | 0 | 0 | 0 | 1 | 3 | 0 | 1 | 4 | 1 | 0 | 0 | 1 | 0 | 0 | 0 | 0 | 0 | 0 | 0 | 1 | 4 | 0 | 0 | 2 | 0 | 0 |

Phytohormon responsive — Plant growth and development — Abiotic and biotic stresses

**Figure S3.** Cis-acting elements of cyclin and CDK genes in promoters.
