# Supplementary material for: Study on the Interactions of Cyclins with CDKs Involved in Auxin Signal during Leaf Development by WGCNA in Populus alba
Source: Int J Mol Sci. 2023 Aug 30;24(17):13445. doi: 10.3390/ijms241713445 (PMC10487486; doi:10.3390/ijms241713445)
Supplement: Supplementary file 1 [file ijms-24-13445-s001.zip › Supplementary Files/Figure S5. The GO and KEGG enrichment of the DEGs by IBA treatment.pdf]

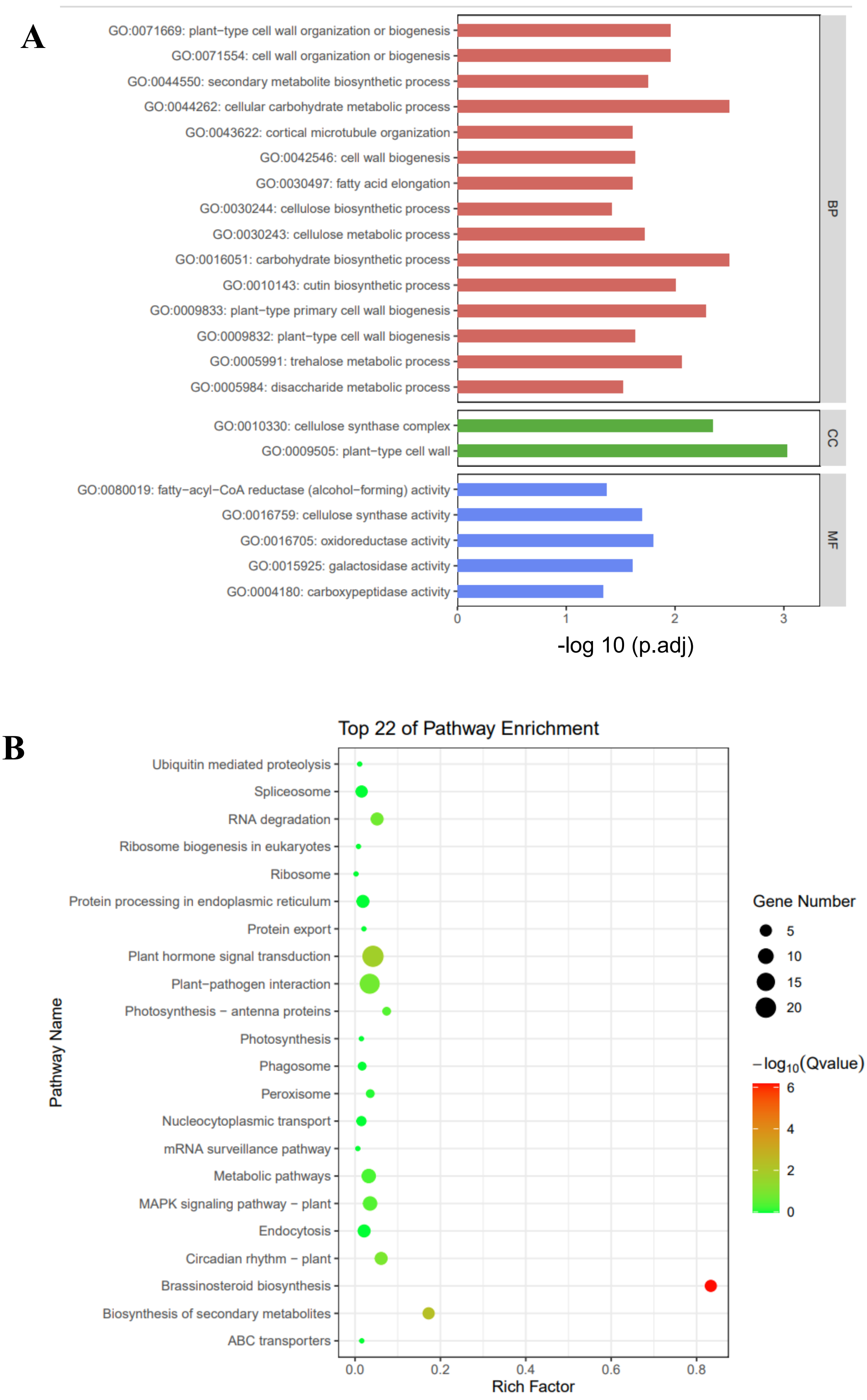

Figure S5. The GO and KEGG enrichment of the DEGs by IBA treatment. **A** GO enrichment of DEGs. **B** KEGG enrichment of DEGs.
