# Supplementary figures and images for: Study on the Interactions of Cyclins with CDKs Involved in Auxin Signal during Leaf Development by WGCNA in Populus alba
Source: Int J Mol Sci. 2023 Aug 30;24(17):13445. doi: 10.3390/ijms241713445 (PMC10487486; doi:10.3390/ijms241713445)

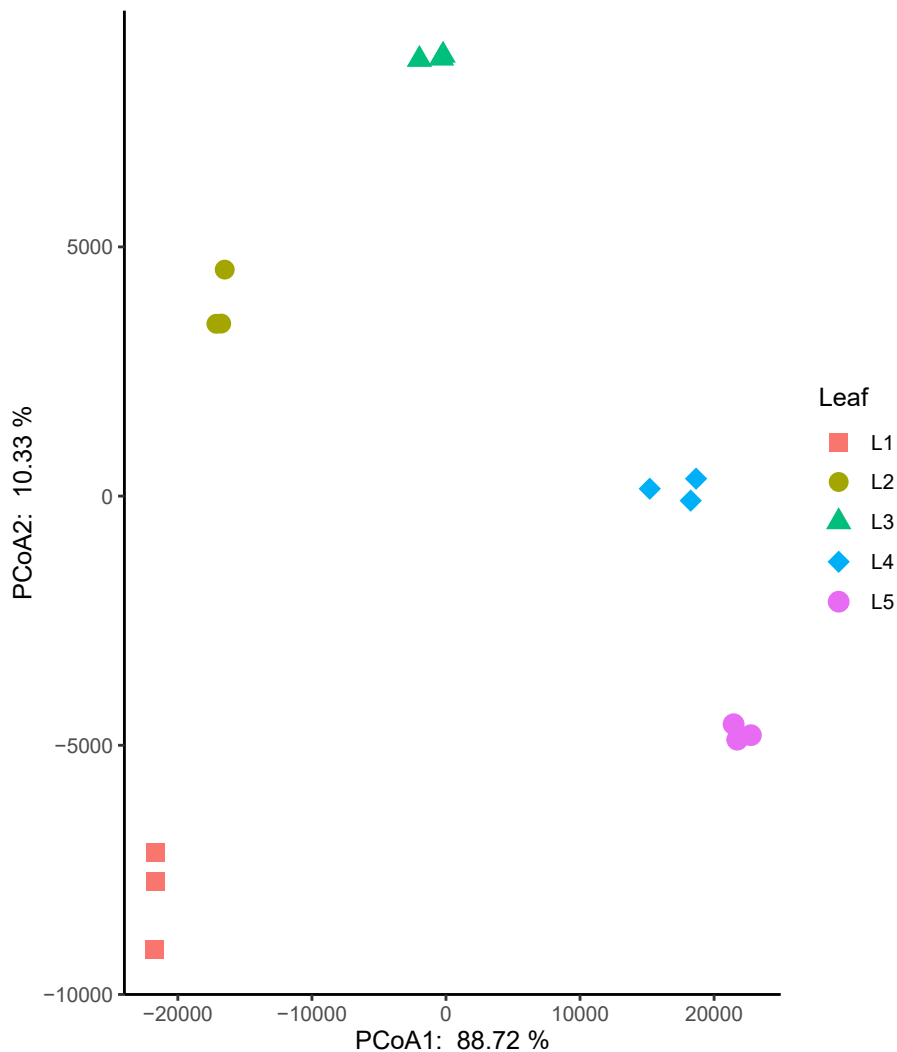

**Figure S10.** PCoA analysis of transcriptome

Supplement: Supplementary file 1 [file ijms-24-13445-s001.zip › Supplementary Files/Figure S10. PCoA analysis of transcriptome.pdf]

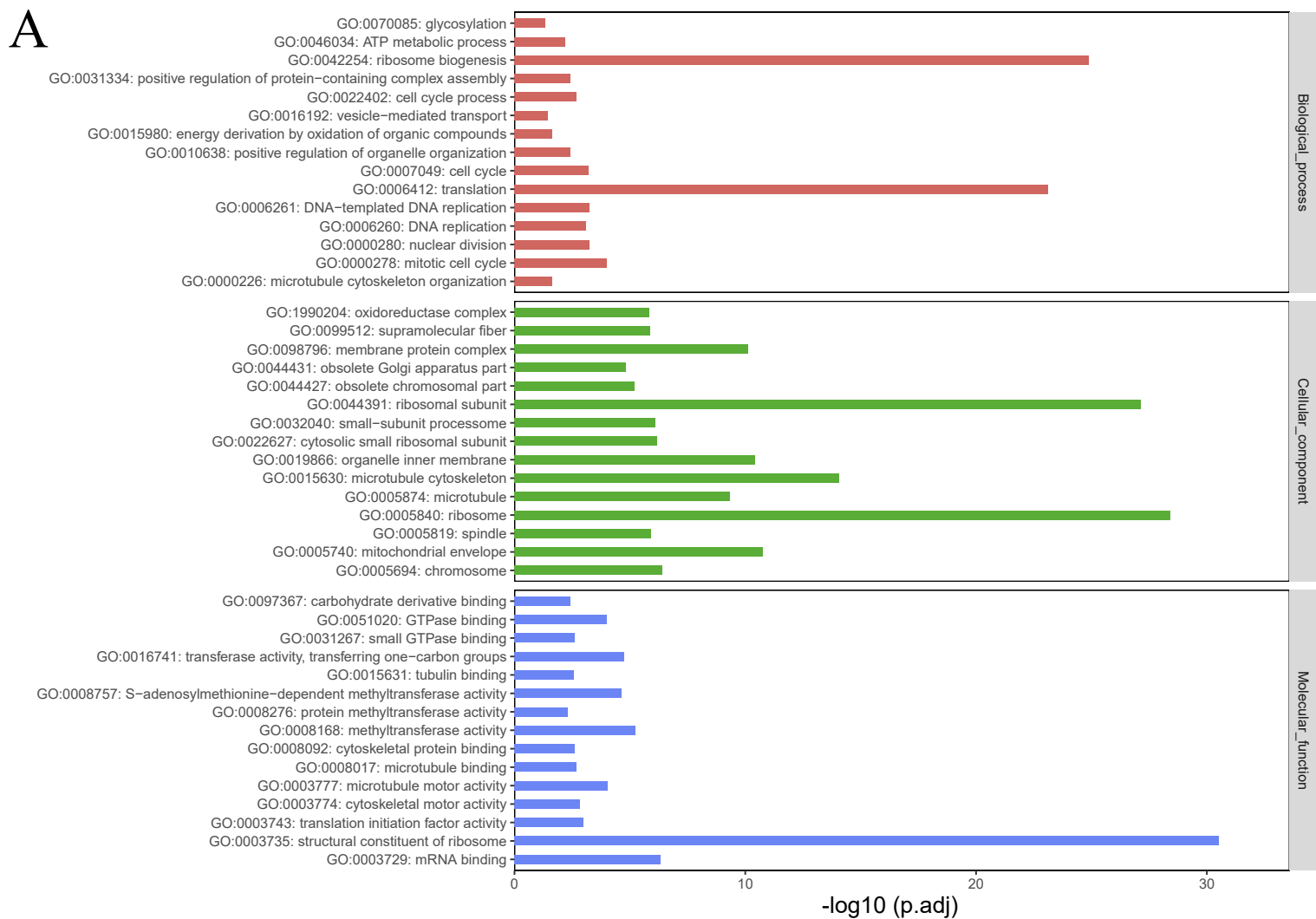

**B**

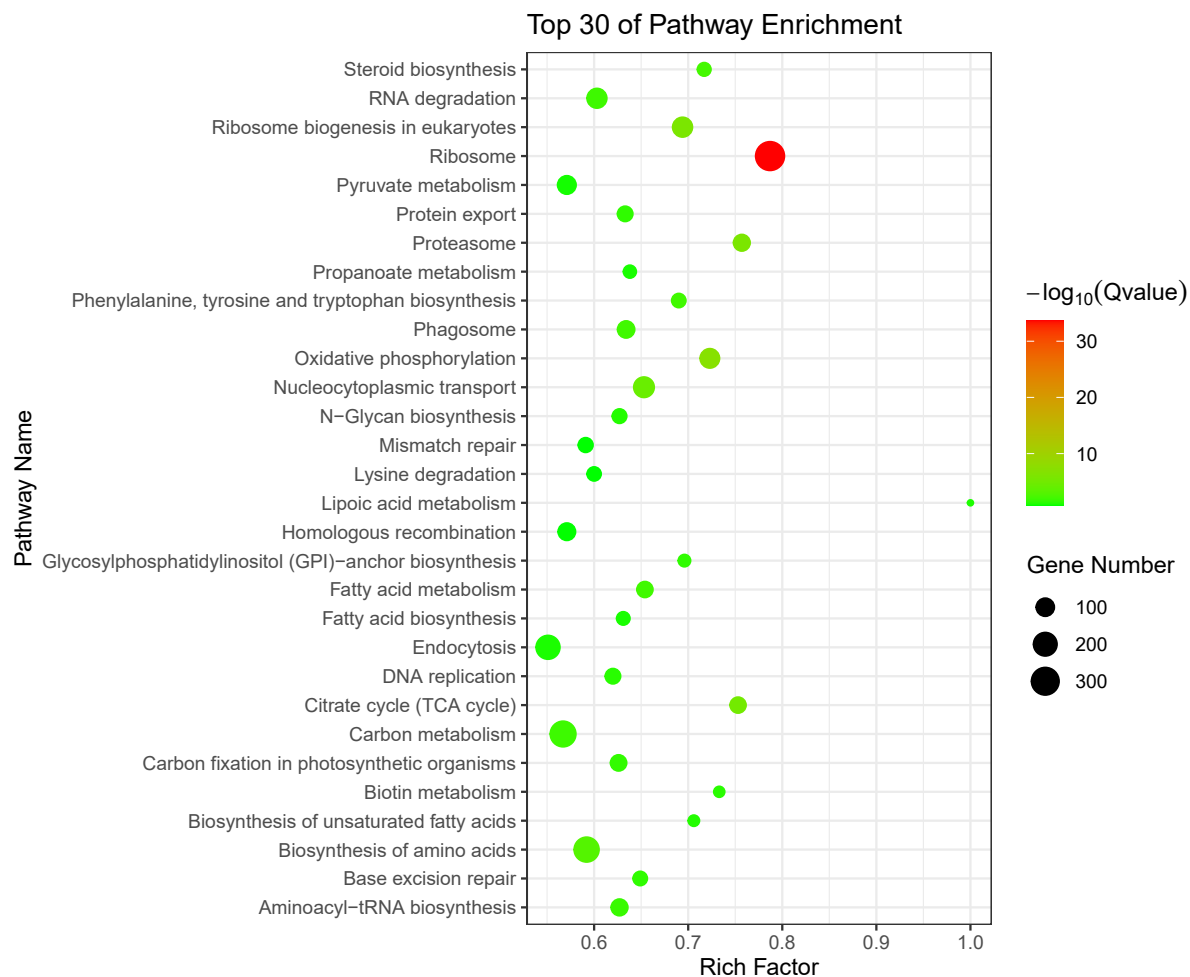

**Figure S11. GO and KEGG enrichment of turquoise module.**  
**A** GO plot. **B** KEGG plot.

Supplement: Supplementary file 1 [file ijms-24-13445-s001.zip › Supplementary Files/Figure S11. GO and KEGG enrichment of turquoise module.pdf]

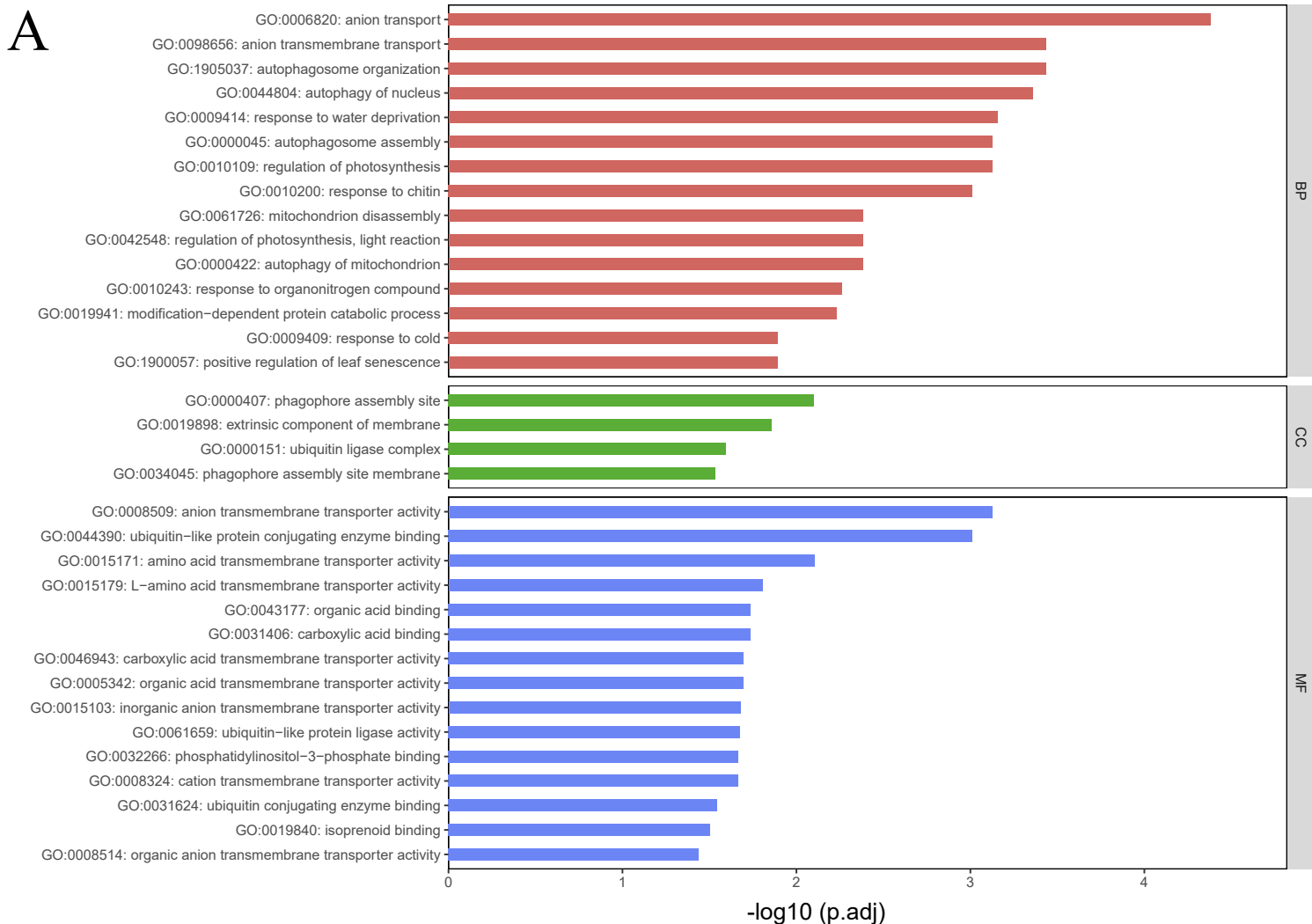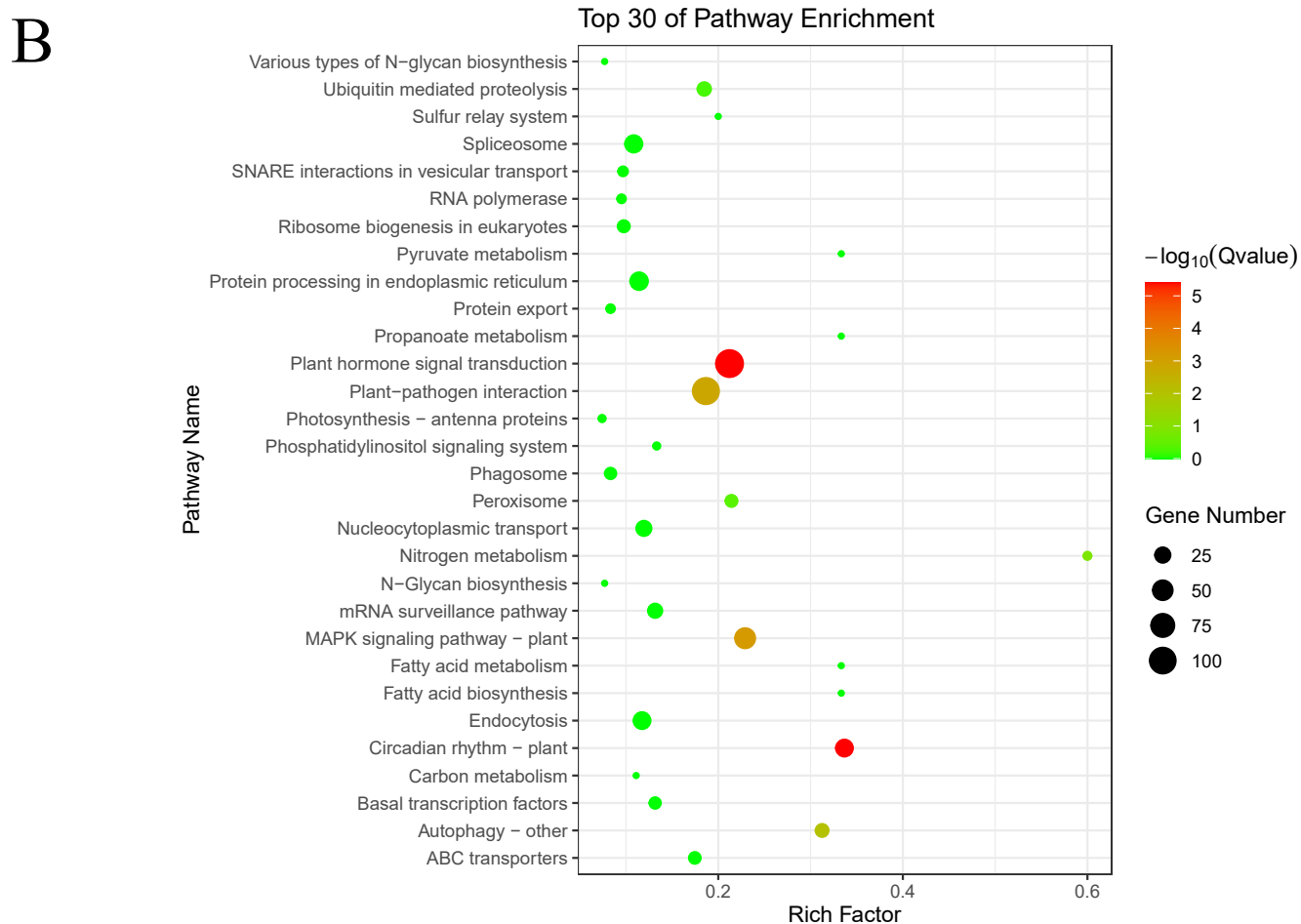

**Figure S12. GO and KEGG enrichment of blue module.**  
**A** GO plot. **B** KEGG plot

Supplement: Supplementary file 1 [file ijms-24-13445-s001.zip › Supplementary Files/Figure S12. GO and KEGG enrichment of blue module.pdf]

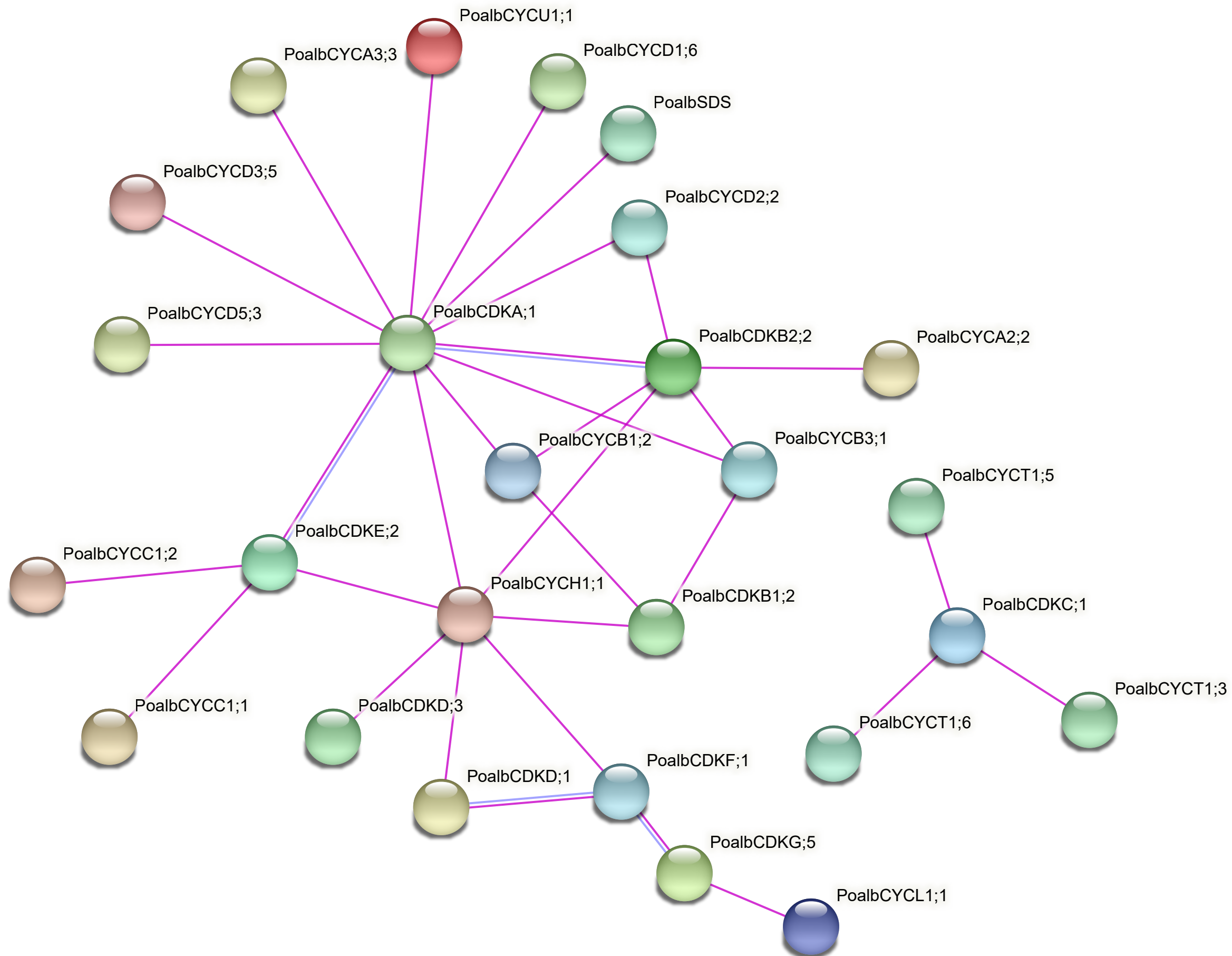

**Figure S4.** The interaction network of cyclins and CDKs

Supplement: Supplementary file 1 [file ijms-24-13445-s001.zip › Supplementary Files/Figure S4. The interaction network of cyclins and CDKs.pdf]

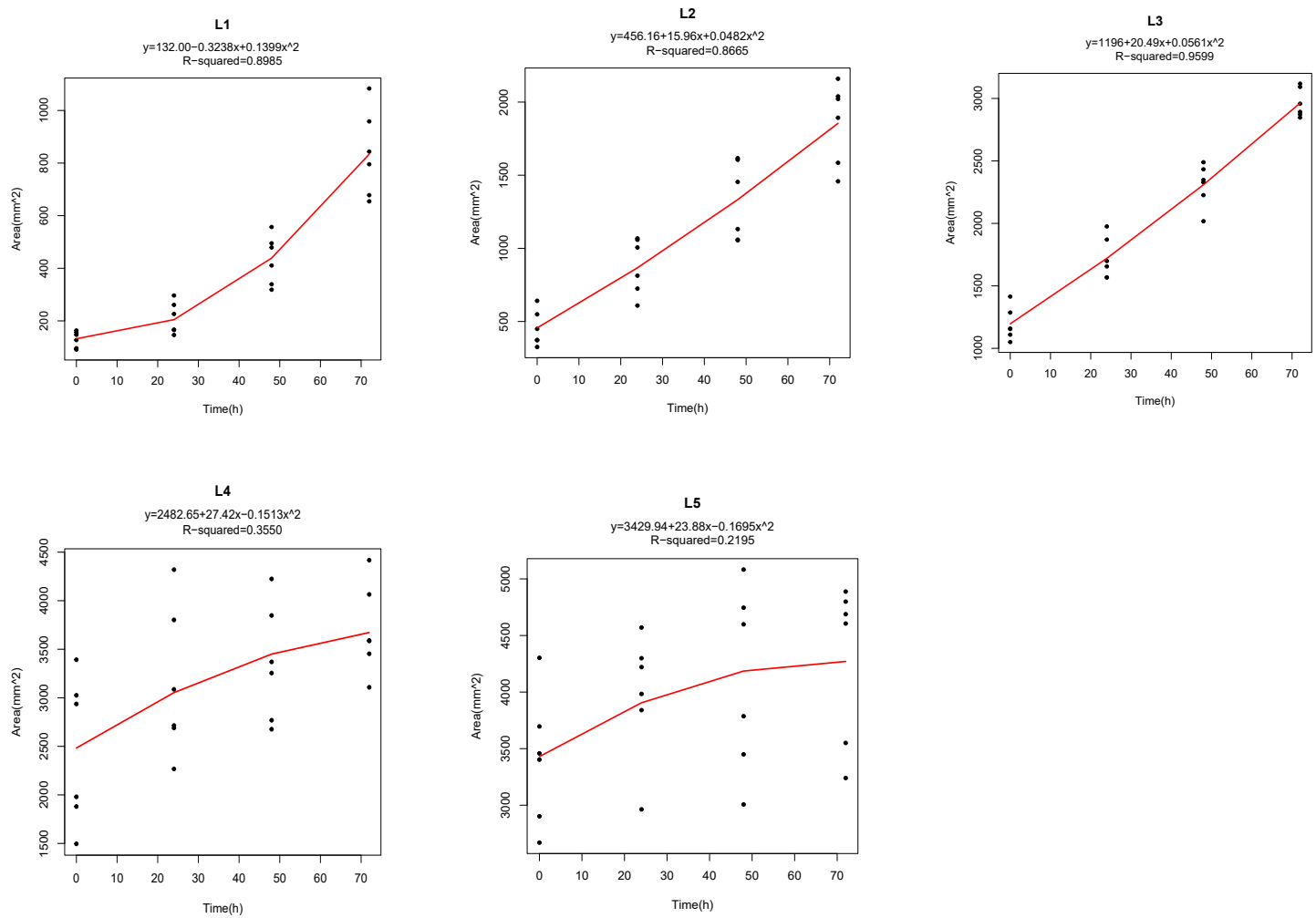

**Figure S6.** Univariate binomial fitting curve. The R-squared indicated the explained data.

Supplement: Supplementary file 1 [file ijms-24-13445-s001.zip › Supplementary Files/Figure S6. Univariate binomial fitting curve.pdf]

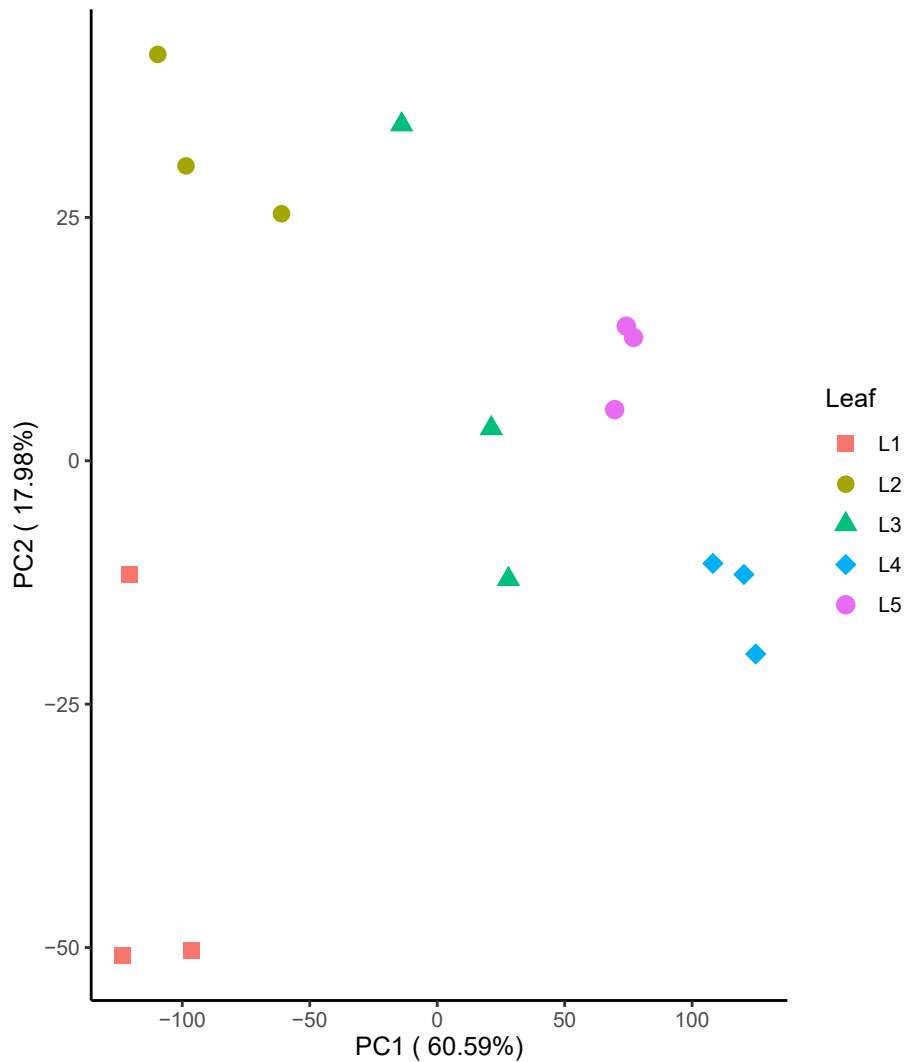

**Figure S8.** PCA analysis of hormones

Supplement: Supplementary file 1 [file ijms-24-13445-s001.zip › Supplementary Files/Figure S8. PCA analysis of hormones.pdf]

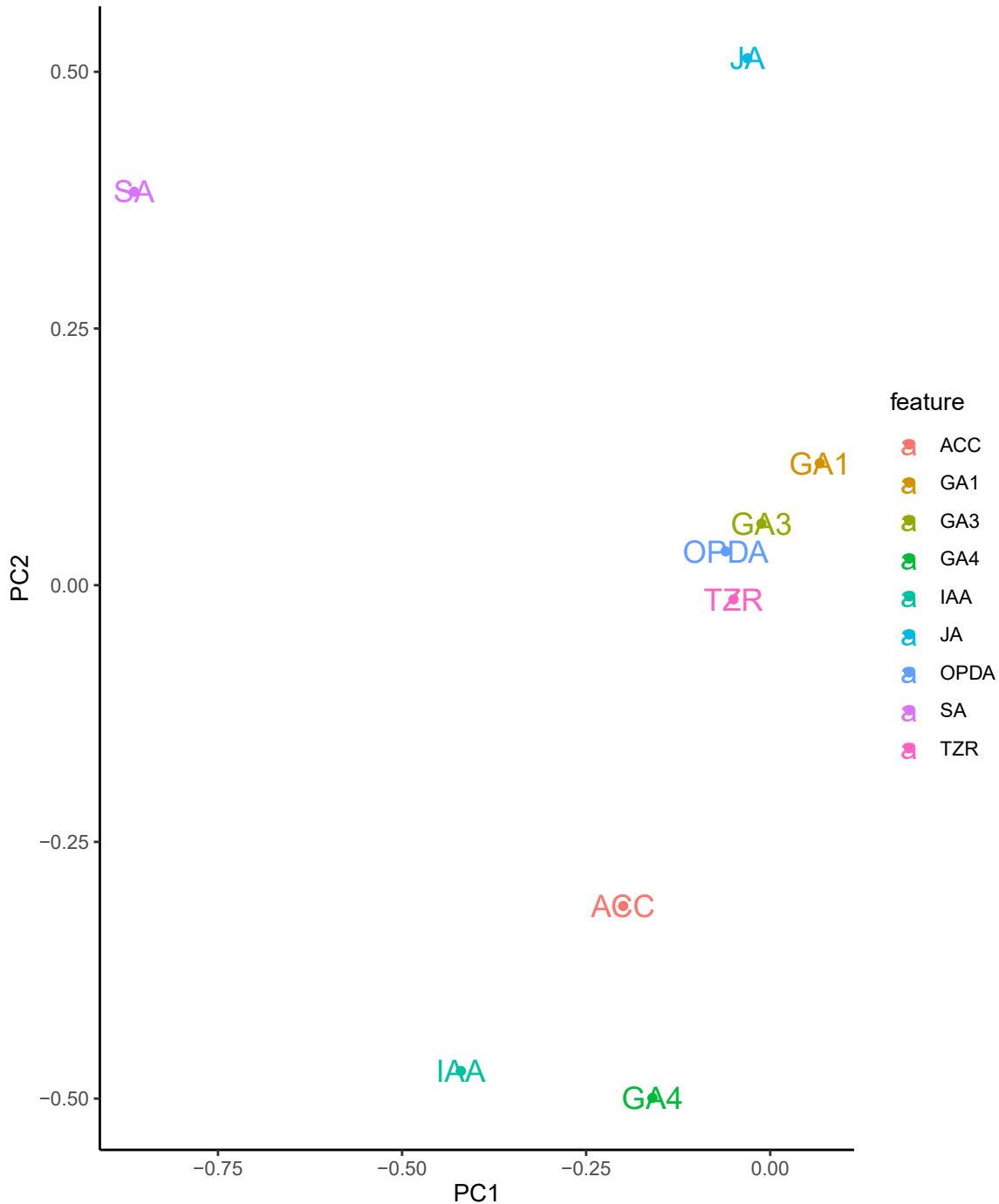

**Figure S9.** Contribution of PCA analysis

Supplement: Supplementary file 1 [file ijms-24-13445-s001.zip › Supplementary Files/Figure S9. Contribution of PCA analysis.pdf]
